# Improving CRISPR guide design with consensus approaches

**DOI:** 10.1186/s12864-019-6291-z

**Published:** 2019-12-24

**Authors:** Jacob Bradford, Dimitri Perrin

**Affiliations:** School of Electrical Engineering and Computer Science, Science and Engineering Faculty, Queensland University of Technology (QUT), 2 George Street, Brisbane, QLD 4000 Australia

**Keywords:** CRISPR, Guide design, Consensus

## Abstract

**Background:**

CRISPR-based systems are playing an important role in modern genome engineering. A large number of computational methods have been developed to assist in the identification of suitable guides. However, there is only limited overlap between the guides that each tool identifies. This can motivate further development, but also raises the question of whether it is possible to combine existing tools to improve guide design.

**Results:**

We considered nine leading guide design tools, and their output when tested using two sets of guides for which experimental validation data is available. We found that consensus approaches were able to outperform individual tools. The best performance (with a precision of up to 0.912) was obtained when combining four of the tools and accepting all guides selected by at least three of them.

**Conclusions:**

These results can be used to improve CRISPR-based studies, but also to guide further tool development. However, they only provide a short-term solution as the time and computational resources required to run four tools may be impractical in certain applications.

## Background

Wild-type CRISPR (Clustered Regularly Interspaced Short Palindromic Repeats) act as an adaptable immune system in archaea and bacteria [1]. The process by which the CRISPR system provides immunity has three main steps [2]:

1. a DNA snippet from an invading phage is obtained and stored within the CRISPR array, making a memory of past viral infection;
2. the CRISPR region is expressed and matured to produce duplicates of previously obtained DNA snippets (or *guides*);
3. a guide binds with an RNA-guided endonuclease (e.g. Cas9, in the case of *S. pyogenes*) to enable site-specific cleavage through homology between the guide and the DNA sequence of the invading phage.

This last step is the mechanism by which CRISPR can be used in a genome engineering context, where a synthetic guide is supplied. CRISPR-based systems have been used for a number of such applications [3–5]. However, guide design is not trivial. The efficacy and specificity of guides are crucial factors. For this reason, computational techniques have been developed to identify and evaluate candidate CRISPR-Cas9 guides.

In a benchmark of the leading guide design tools, we previously noted the limited overlap between the guides that each tool selects [6]. In the long term, this justifies the development of a new generation of tools, which will combine the best features of existing tools and provide a more exhaustive and more reliable selection of guides. In the meantime, this poses an important question: is it possible to combine the results of existing tools to improve guide selection?

To answer this question, we analysed the output of nine distinct guide design tools on experimental data and investigated whether the consensus between some or all of the tools would lead to a better set of guides.

## Results

### Individual tools

We tested each tool on two datasets (namely *Wang* and *Doench*), which contains guides for which the efficiency has been experimentally assessed. For each dataset, we considered two recall thresholds: 0.2 and 0.5 (see Methods).

First, the performance of each tool was measured individually. It was found that most tools provide useful results given the constraints of each dataset. For the Doench dataset, a lower precision is observed. This is consistent with the portion of efficient guides in Doench being smaller than in Wang. The results are summarised in Table 1.

**Table 1:** Results for individual tools

	Wang				Doench			
Tool name *n*	Accepted	Precision	Recall	NPV	Accepted	Precision	Recall	NPV
Cas-Designer	206	0.612	0.172	0.372	668	0.210	0.377	0.803
SSC	632	0.851*	0.736	0.641	1056	0.277	0.787	0.899
PhytoCRISP-Ex	348	0.764	0.364	0.434	524	0.235	0.332	0.812
TUSCAN	737	0.715	0.721	0.528	1390	0.245*	0.919	0.934
sgRNAScorer2	484	0.833	0.551	0.521	893	0.270	0.650	0.863
mm10db	330	0.652	0.294	0.385	384	0.227	0.235	0.805
CHOPCHOP	273	0.843	0.315	0.441	537	0.294	0.426	0.837
CHOPCHOP-Xu	638	0.853*	0.744	0.648	1061	0.197	0.563	0.792
CHOPCHOP-MM	338	0.716	0.331	0.412	761	0.191	0.391	0.791
WU-CRISPR	286	0.818	0.320	0.437	366	0.511*	0.504	0.875
FlashFry	141	0.844	0.163	0.405	210	0.586*	0.332	0.848

^*^indicates tool was trained using this dataset

**Fig. 1:**
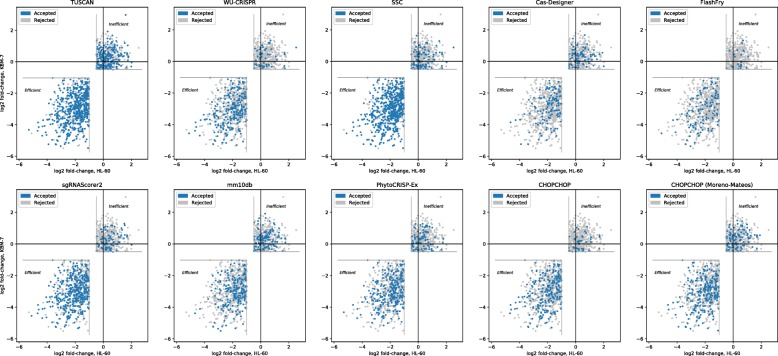
Results for individual tools on the Wang dataset

When testing on the Wang dataset and seeking a recall of 0.2, CHOPCHOP achieved the highest precision: 0.843. When seeking a recall of at least 0.5, sgRNAScorer2 achieved the highest precision on this dataset: 0.833. The guides selected by each tool are shown in Fig. 1.

**Fig. 2:**
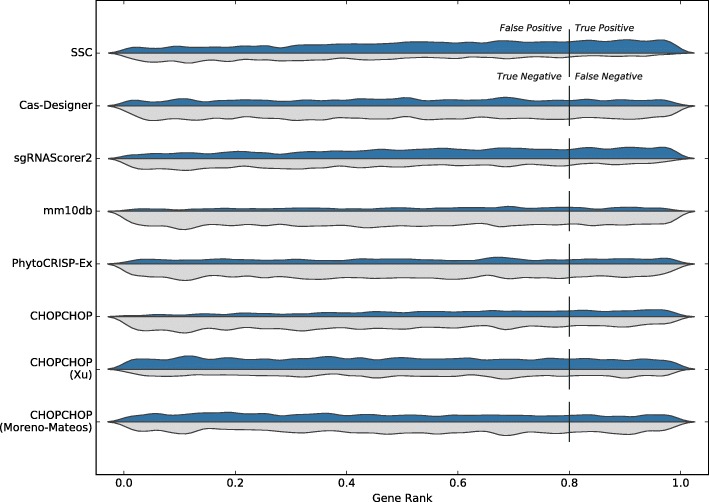
Results for individual tools on the Doench dataset. The blue distribution shows the number of guides accepted, and the grey distribution shows the number of guides rejected. The vertical marker at 0.8 shows the threshold used to determine efficiency; guides with a gene rank score greater than this were deemed experimentally efficient [19]

When testing on the Doench dataset, CHOPCHOP again achieved the best precision for a recall of 0.2, at 0.294. When seeking a recall of at least 0.5, SSC achieved the highest precision, at 0.277. The distribution of guides accepted and rejected by each tool are shown in Fig. 2.

Next, for tools that rely on a score threshold to reject and accept guides, we considered the impact of that threshold. For most of these tools, it was not possible to find a better configuration: while increasing the threshold increases the precision, it quickly dropped the recall below our target values. The only exception was SSC on the Doench dataset. The optimal solution was to raise the threshold from 0.0 to 0.55 (range is −1.0 to 1.0), which maintained the recall above 0.2 and raised the precision to 0.355. This also improves its performance on the Wang dataset, but SSC uses that dataset for training so it is not relevant. For a recall above 0.5, the optimal threshold for SSC was 0.2, for a precision of 0.300. However, it is important to note that SSC is implemented so that it can only work on small input sequences, and does not do any off-target scoring. It therefore cannot be used as a stand-alone tool, especially when considering entire genomes.

### Simple consensus

The most intuitive way to combine results from separate tools was to only accept guides that have been selected by at least *n* tools.

First, we consider an approach where all tools are included, except if they are trained using the dataset used for tests or if they did not successfully complete at least two tests in the benchmark study [6]. This means that, for, when testing on the Wang dataset, the set considered for the consensus includes: Cas-Designer, WU-CRISPR, FlashFry, sgRNAScorer2, CHOPCHOP, CHOPCHOP-MM, TUSCAN, PhytoCRISP-Ex and mm10db. When testing on the Doench dataset, the set includes: Cas-Designer, sgRNAScorer2, CHOPCHOP, CHOPCHOP-Xu, CHOPCHOP-MM, PhytoCRISP-Ex and mm10db.

The results are shown in Table 2. As can be expected, guides that were selected by many tools were more likely to be efficient. However, a strict intersection of the results from each tool would not be practical: on both datasets, only a handful are identified by all tools. At the other end of the spectrum (i.e. choosing *n*=1), there was a very high recall on both datasets, but this approach had a low precision.

**Table 2:** Consensus when removing models trained on the associated test dataset

	Wang				Doench			
Consensus level *n*	Accepted	Precision	Recall	NPV	Accepted	Precision	Recall	NPV
1	1050	0.663	0.952	0.706	1751	0.205	0.968	0.867
2	857	0.712	0.835	0.612	1472	0.218	0.865	0.865
3	578	0.811	0.642	0.557	947	0.244	0.623	0.843
4	357	0.868	0.424	0.482	490	0.282	0.372	0.828
5	180	0.911	0.224	0.427	136	0.331	0.121	0.809
6	81	0.926	0.103	0.397	29	0.276	0.022	0.800
7	30	0.967	0.040	0.384	3	0.333	0.003	0.799
8	9	0.889	0.011	0.377	-	-	-	-
9	1	1.000	0.001	0.375	-	-	-	-

Tools considered for Wang: Cas-Designer, WU-CRISPR, FlashFry, sgRNAScorer2, CHOPCHOP, CHOPCHOP-MM, TUSCAN, PhytoCRISP-Ex and mm10db. Tools considered for Doench: Cas-Designer, sgRNAScorer2, CHOPCHOP, CHOPCHOP-Xu, CHOPCHOP-MM, PhytoCRISP-Ex and mm10db

As described in Methods, we considered two levels of recall (0.2 and 0.5) that address the needs of specific experimental settings.

If a recall of at least 0.2 is appropriate, the best results on the Wang dataset were obtained for *n*=5, with a precision of 0.911. This is higher than any individual tool. In contexts where a higher recall is needed (0.5), a precision of 0.811 can be achieved with *n*=3.

On the Doench dataset, for a recall of 0.2, a precision of 0.282 was achieved with *n*=4. This is higher than any of these tools taken individually, apart from CHOPCHOP. For a recall of 0.5, a precision of 0.244 was achieved with *n*=3.

Cas-Designer had the lowest overall performance (lowest precision and second-lowest recall on Wang, third-lowest precision on Doench). Excluding Cas-Designer and repeating the consensus approach for the remaining tools produced similar, but improved, results. The highest precision with acceptable recall is now 0.925 on Wang and 0.303 on Doench, and the highest precision with high recall is now 0.831 on Wang and 0.260 on Doench. Continuing this approach by excluding a second tool was not convincing, but motivates further exploration with smaller list of tools.

**Fig. 3:**
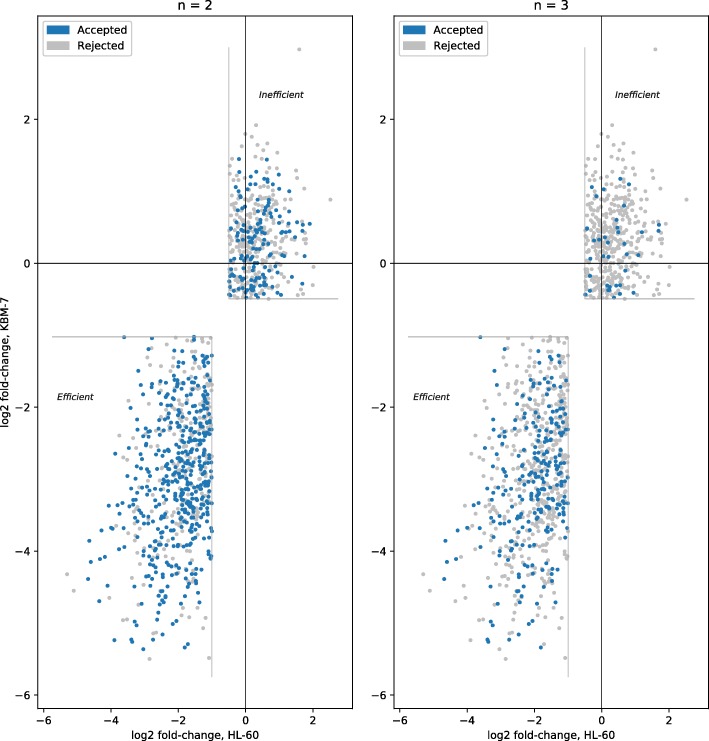
Consensus, on the Wang dataset, when accepting guides selected by at least *n* tools (except those models trained on any of the test data and poor performing tools): Cas-Designer, sgRNAScorer2, CHOPCHOP, CHOPCHOP-MM, PhytoCRISP-Ex, mm10db

**Fig. 4:**
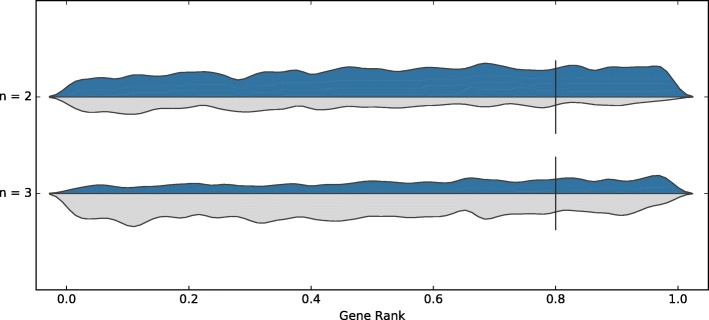
Consensus, on the Doench dataset, when accepting guides selected by at least *n* tools (except those models trained on any of the test data and poor performing tools): Cas-Designer, sgRNAScorer2, CHOPCHOP, CHOPCHOP-MM, PhytoCRISP-Ex, mm10db

We also considered when tools trained on either dataset were removed. The tools used for the consensus are then Cas-Designer, sgRNAScorer2, CHOPCHOP, CHOPCHOP-MM, PhytoCRISP-Ex and mm10db. The results from this approach are shown in Table 3. The precision is comparable, but the recall decreases slightly. The distribution of guides are shown in Figs. 3 and 4 for both datasets.

**Table 3:** Consensus: accepting guides selected by at least *n* tools (except those models trained on the test data and poor performing tools)

	Wang				Doench			
Consensus level *n*	Accepted	Precision	Recall	NPV	Accepted	Precision	Recall	NPV
1	953	0.681	0.888	0.620	1662	0.209	0.935	0.866
2	607	0.763	0.633	0.523	1214	0.236	0.771	0.864
3	284	0.870	0.338	0.453	642	0.280	0.485	0.841
4	102	0.912	0.127	0.402	207	0.324	0.181	0.814
5	30	0.900	0.037	0.382	39	0.333	0.035	0.801
6	3	1.000	0.004	0.376	3	0.333	0.003	0.799

Tools considered here: Cas-Designer, sgRNAScorer2, CHOPCHOP, CHOPCHOP-MM, PhytoCRISP-Ex and mm10db

### Design-specific consensus

**Fig. 5:**
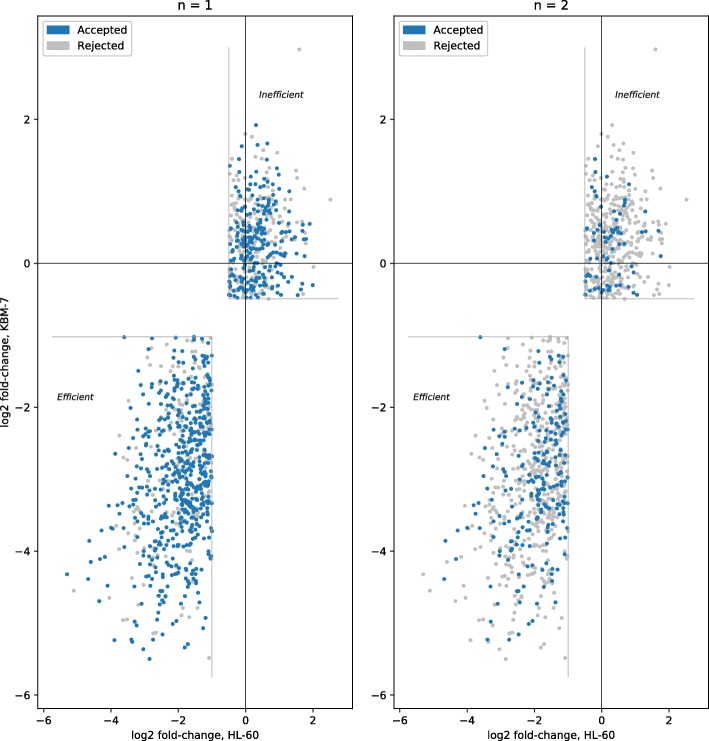
Consensus, on the Wang dataset, between procedural methods: Cas-Designer, CHOPCHOP, PhytoCRISP-Ex, mm10db

**Fig. 6:**
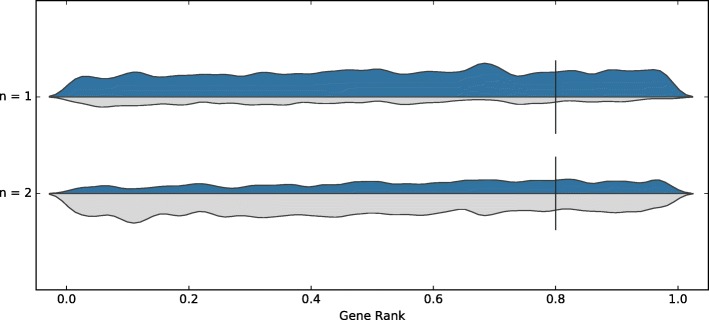
Consensus, on the Doench dataset, between procedural methods: Cas-Designer, CHOPCHOP, PhytoCRISP-Ex, mm10db

Next, we explored whether the design approach had any impact; we grouped the machine-learning (ML) methods, and the procedural methods. The results on the consensus of procedural methods are shown in Table 4, Figs. 5 and 6. A consensus approach based solely on procedural methods does not appear to be useful.

**Table 4:** Consensus between procedural methods

	Wang				Doench			
Consensus level *n*	Accepted	Precision	Recall	NPV	Accepted	Precision	Recall	NPV
1	790	0.696	0.752	0.522	1411	0.220	0.838	0.861
2	299	0.769	0.315	0.424	595	0.259	0.415	0.826
3	61	0.853	0.071	0.387	97	0.392	0.102	0.809
4	7	0.714	0.007	0.375	10	0.500	0.014	0.800

Cas-Designer, CHOPCHOP, PhytoCRISP-Ex, mm10db

For ML methods, we followed the same strategy as above, and removed tools trained on the data used in our tests. The results are summarised in Table 5.

**Table 5:** Consensus between machine-learning methods, removing models trained on the associated test dataset

	Wang			Doench				
Consensus level *n*	Accepted	Precision	Recall	NPV	Accepted	Precision	Recall	NPV
1	927	0.695	0.881	0.641	1655	0.216	0.965	0.930
2	624	0.793	0.677	0.567	1242	0.241	0.806	0.880
3	295	0.881	0.356	0.461	653	0.254	0.447	0.827
4	120	0.892	0.146	0.405	221	0.290	0.173	0.811
5	20	0.950	0.026	0.380	-	-	-	-

Tools considered for Wang: sgRNAScorer2, CHOPCHOP-MM, WU-CRISPR, FlashFry and TUSCAN. Tools considered for Doench: sgRNAScorer2, SSC, CHOPCHOP-MM and CHOPCHOP-Xu

For the Wang dataset, this means that we considered the consensus between sgRNAScorer2, CHOPCHOP-MM, WU-CRISPR, FlashFry and TUSCAN. Given a recall of at least 0.2, the approach had a precision of 0.881 when *n*=3. For a recall of at least 0.5, the approach had a precision of 0.793 when *n*=2.

For the Doench dataset we considered sgRNAScorer2, SSC, CHOPCHOP-MM and CHOPCHOP-Xu. Here, aiming for a recall above 0.2, the best precision was 0.254 (for *n*=3). With *n*=4, it is possible to reach a precision of 0.290, but the recall is only 0.173.

Only considering ML tools that are not trained on either dataset is not useful, as there are only two such methods (sgRNAScorer2 and CHOPCHOP-MM).

### Optimal consensus

**Fig. 7:**
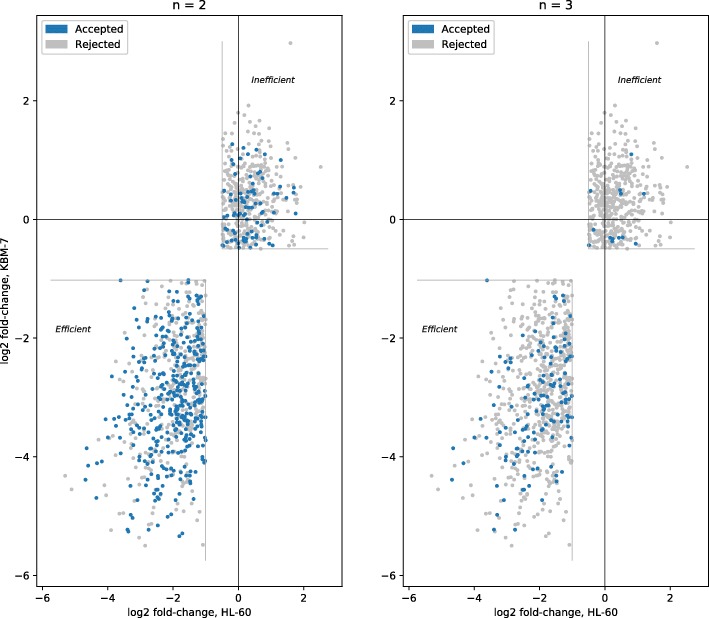
Consensus, on the Wang dataset, when optimising for both datasets (excluding models trained on test data, excluding poor performing tools, no more than five tools, recall approx. 20%): sgRNAScorer2, CHOPCHOP, PhytoCRISP-Ex, mm10db

**Fig. 8:**
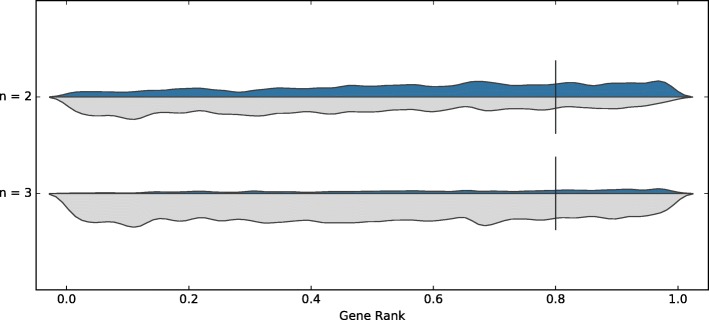
Consensus, on the Doench dataset, when optimising for both datasets (excluding models trained on test data, excluding poor performing tools, no more than five tools, recall approx. 20%): sgRNAScorer2, CHOPCHOP, PhytoCRISP-Ex, mm10db

Based on the earlier results, we tried to identify the best set of tools to use for consensus, with only the same two constraints as above: the tool should not have been trained on the dataset used for testing, and it should have completed at least two tests in the benchmark. Here, we optimise for the highest possible precision, while maintaining a recall of approximately 0.2. The best approach was obtained using sgRNAScorer2, CHOPCHOP, PhytoCRISP-Ex and mm10db; the results are shown in Table 6, Figs. 7 and 8. If accepting guides selected by at least three of these four tools, we obtained a precision of 0.912 (recall 0.185) and 0.356 (recall 0.216) for Wang and Doench, respectively. These results outperform those from individual tools or from the simple consensus approach.

**Table 6:** Consensus when optimising for both datasets (excluding models trained on test data, excluding poor performing tools, no more than five tools, recall approx. 20%)

	Wang				Doench			
Consensus level *n*	Accepted	Precision	Recall	NPV	Accepted	Precision	Recall	NPV
1	811	0.724	0.803	0.598	1327	0.229	0.819	0.870
2	453	0.817	0.506	0.496	762	0.280	0.574	0.854
3	148	0.912	0.185	0.416	225	0.356	0.216	0.820
4	23	0.957	0.030	0.381	24	0.500	0.032	0.802

Tools considered here: sgRNAScorer2, CHOPCHOP, PhytoCRISP-Ex, mm10db

One limitation is that this approach is using two of the slowest tools (sgRNAScorer2 and PhytoCRISP-Ex), as per our earlier benchmark [6]. It is possible to be computationally more efficient by excluding PhytoCRISP-Ex, at a cost in terms of precision, but still outperforms individual tools: 0.857 for Wang (recall 0.360) and 0.293 for Doench (recall 0.453), with *n*=2.

## Discussion

Our results clearly show that there is scope for further development of CRISPR guide design methods. While most tools taken in isolation tend to produce high-quality guides, the lack of overlap between their results is striking. This has two main implications.

First, it means that using a single design tool would lead to some quality guides being incorrectly rejected. While most tools report enough guides for most applications, this can be an issue for contexts where the search region is small: only SSC, Tuscan and sgRNAScorer2 have a high recall on both datasets. Using a single design tool would also lead to some lower quality guides still being incorrectly selected. None of the tools had a precision over 0.85 on Wang or over 0.3 on Doench. The design strategy (machine learning vs. procedural approaches) did not make a difference, whether we considered individual tools, tools grouped by approach, or even the consensus between the approaches.

Second, it means that further development is needed. We showed that the consensus between four tools (sgRNAScorer2, CHOPCHOP, PhytoCRISP-Ex and mm10db) can be used to generate a set of guides where up to 91.2% are efficient (on the Wang dataset), while still maintaining appropriate recall. However, this comes with several downsides: (i) the time required to prepare four tools and datasets in the various formats required, and to perform the consensus analysis; and (ii) the limitations of some of these tools in terms of compute resources and scalability. In particular, we previously reported that two of the tools (PhytoCRISP-Ex and sgRNAScorer2) did not scale to exhaustive searches on large genomes [6].

When taking speed into account and trying to only use tools that have been shown to scale to large genomes, a consensus approach can still generate useful results. Here, we achieved precision of up to 0.852. However, this still does not remove the need to run multiple tools.

Rather than combining the output of tools, future work will need to focus on integrating and optimising the most useful features of these tools.

## Conclusions

A number of tools have been developed to facilitate CRISPR-based genome engineering. Most of them perform adequately, but the overlap between their results is strikingly limited. In this study, we investigated whether existing tools can be combined to produce better sets of guides. We found that consensus approaches were able to outperform all individual tools.

In particular, we found that, by considering four tools (sgRNAScorer2, CHOPCHOP, PhytoCRISP-Ex and mm10db) and accepting all guides selected by at least three of them, we were able to generate a set of guides that contained over 91.2% of efficient guides.

These results provides a short-term solution for guide selection. They also emphasise the need for new methods. Running four separate tools is computationally expensive. Future tools will be able to directly combine the most useful features of these methods, and produce high-quality guides in a reasonable amount of time.

## Methods

### Guide design tools

We previously benchmarked the leading open-source tools for guide design for the *Streptococcus pyogenes*-Cas9 (SpCas9) nuclease, to evaluate them in terms of computational performance as well as in terms of the guides they produce [6]. Some of these tools do not filter guides based on anticipated efficiency, for instance because they focus on off-target predictions.

Here, we therefore focused on nine tools that actively filter or score candidate guides: CHOPCHOP [7], SSC [8], WU-CRISPR [9], Cas-Designer [10], mm10 CRISPR Database – mm10db [11], PhytoCRISP-Ex [12], sgRNA Scorer 2.0 [13], FlashFry [14], and TUSCAN [15]. CHOPCHOP, in default mode, provides a flag indicating whether a guanine is present at position 20 (CHOPCHOP-G20), and also provides models from [8] (CHOPCHOP-Xu) and [16] (CHOPCHOP-MM). All tools are available for download, with access details summarised in Table 7.

**Table 7:** Tools selected in this study

Tool	Availability	Approach	Language	Training Dataset
Cas-Designer (8 May 2018)	Tool website	Procedural	Python	
mm10 CRISPR Database (*92d208c*)	GitHub	Procedural	Python / C	
PhytoCRISP-Ex (v1.0)	Tool website	Procedural	Perl / Bash	
WU-CRISPR (*7107166*)	GitHub	ML	Perl	*Doench*
sgRNA Scorer 2.0	Tool website	ML	Python	Other
FlashFry (2 July 2018)	GitHub	ML	Java	*Doench* and one other
TUSCAN (offline edition - 24 January 2019)	By request	ML	Python	*Doench* and four others
SSC (v0.1)	Sourceforge	ML	C	*Wang* and four others
CHOPCHOP (*384743c*)	BitBucket	Both	Python	*Wang* or *Doench* or other

ML is Machine Learning; TUSCAN and SSC combined the listed training datasets as one; CHOPCHOP and FlashFry provide each of the listed efficacy scoring models; CHOPCHOP offers efficacy scoring via a procedural approach. The italicised text in the ‘Tool’ column is the git hash to identify which version of the tool was used. Similarly, for the date obtained or version number indicated

There is a broad range of approaches. Some tools are using machine-learning models, while others take a procedural approach to implement specific biological rules. Within the latter group, the rules also vary between tools. They can include considerations such as avoiding poly-thymine sequences [17], rejecting guides with inappropriate GC-content [18], or considering the secondary structure of the guide RNA. Because of the different approaches taken by the developers, it can be expected that each tool would produce different guides.

For tools that produce a score and require a threshold to accept or reject a guide, we used the recommended where available. The values we used are: 0.5 for FlashFry, 70 for Cas-Designer, 50 for WU-CRISPR, 0.55 for CHOPCHOP-MM, and 0 for SSC, CHOPCHOP-Xu and sgRNAScorer2. Given that our objective is to investigate how existing tools may complement each other, we did not try to change these thresholds, or to improve any of the filtering or scoring of any tool.

### Experimental data

There is not one tool that can be considered as the gold standard to compare performance. Instead, we use two collections of guides for which experimental validation data is available, collated by [18] and [19]. We refer to these datasets as the Wang and Doench datasets, respectively. The Wang dataset pre-processed as in [8] contains 1169 guides used in screening experiments of two human cells lines; 731 were deemed to be ‘efficient’ based on analysis of the gene knock-outs. The Doench dataset contains 1841 guides from nine mouse and human transcripts, with 372 of the guides deemed to be ‘efficient’. When comparing a consensus approach across the two datasets, a lower precision was observed for Doench than Wang. This is expected due to the higher threshold used to determine guide efficacy.

We constructed an artificial sequence that contains these guides, interspaced by 50 Ns to ensure that unexpected overlapping targets cannot be detected. We also created all the files required by any of the tools: custom annotation file (derived from the *refGene* table available via UCSC), 2bit compression file, Bowtie and Bowtie2 indexes, and Burrows-Wheeler Aligner file.

### Evaluation metrics

For each tool (or combination of tools), we classified a guide as:

- A true positive (TP) if the method correctly classified the guide as being efficient;
- A false positive (FP) if it was selected as a good guide but the data shows it to be inefficient;
- A true negative (TN) if the guide was correctly identified as being inefficient;
- A false negative (FN) if it was incorrectly discarded.

Based on this, we were able to calculate the precision (Eq. 1) and recall (Eq. 2) for each tool or combination of tools. The precision gives us how many guides classified as efficient actually were efficient, while the recall tells us how many of the efficient guides were correctly selected. We also considered the negative predictive value (NPV, Eq. 3), which tells us how confident we can be that a rejected guide really would be inefficient.

1 $$ Precision = TP/(TP+FP)   $$

2 $$ Recall = TP/(TP+FN)   $$

3 $$ NPV = TN/(TN+FN)   $$

All these metrics range from 0 to 1, with 1 being best. An ideal guide design tool would obviously have a perfect precision and recall (which would also imply *NPV*=1), but there are not necessarily equally important. In the context of CRISPR-based gene editing, there are possible target sites: more than 245 million in the entire mouse genome, and typically dozens per gene. Even using strategies that require multiple guides, e.g. triple-targeting for gene knock-outs [11], only a handful of efficient targets are needed for each gene of interest. As a result, a perfect recall is less important than a high precision. In this paper, we set a recall of 0.2, meaning that at approximately 20% of the efficient guides are identified. For some applications that are more restricted in terms of target location, such as CRISPR-mediated activation of a promoter or enhancer [20], it may be appropriate to choose a higher recall. Here, we set it at 0.5.

## Data Availability

The datasets used in this study are available from [8] (in their Supplementary Table 1) and [19] (in their Supplementary Table 7). The guide design tools that are used are all available from their respective authors (with access details shown in Table 7).

## Abbreviations

Cas9: CRISPR-associated protein 9
CRISPR: Clustered regularly interspaced short palindromic repeats
ML: machine learning
SpCas9: *Streptococcus pyogenes*-Cas9
